# Supplementary figures and images for: Cas9 degradation in human cells using phage anti-CRISPR proteins
Source: PLoS Biol. 2023 Dec 8;21(12):e3002431. doi: 10.1371/journal.pbio.3002431 (PMC10732428; doi:10.1371/journal.pbio.3002431)

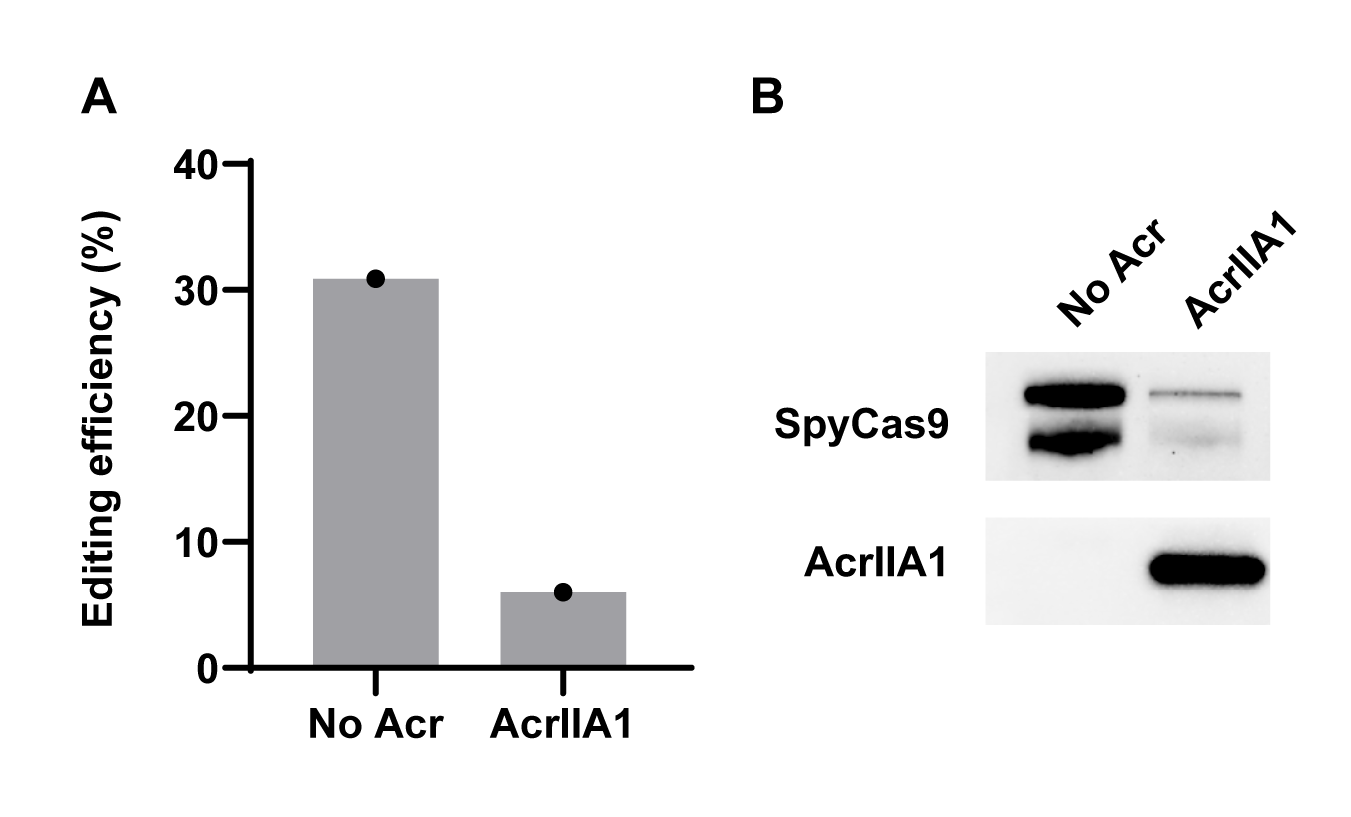

Supplement: S1 Fig — (A) Editing by SpyCas9 of the HBB gene in Hep G2 human liver cells. Bars represent the mean of biological replicates (dots). Underlying data can be found in S1 Data. (B) AcrIIA1 degrades SpyCas9 in Hep G2 cells. Western blot is representative of triplicate. (TIF) [file pbio.3002431.s001.tif]

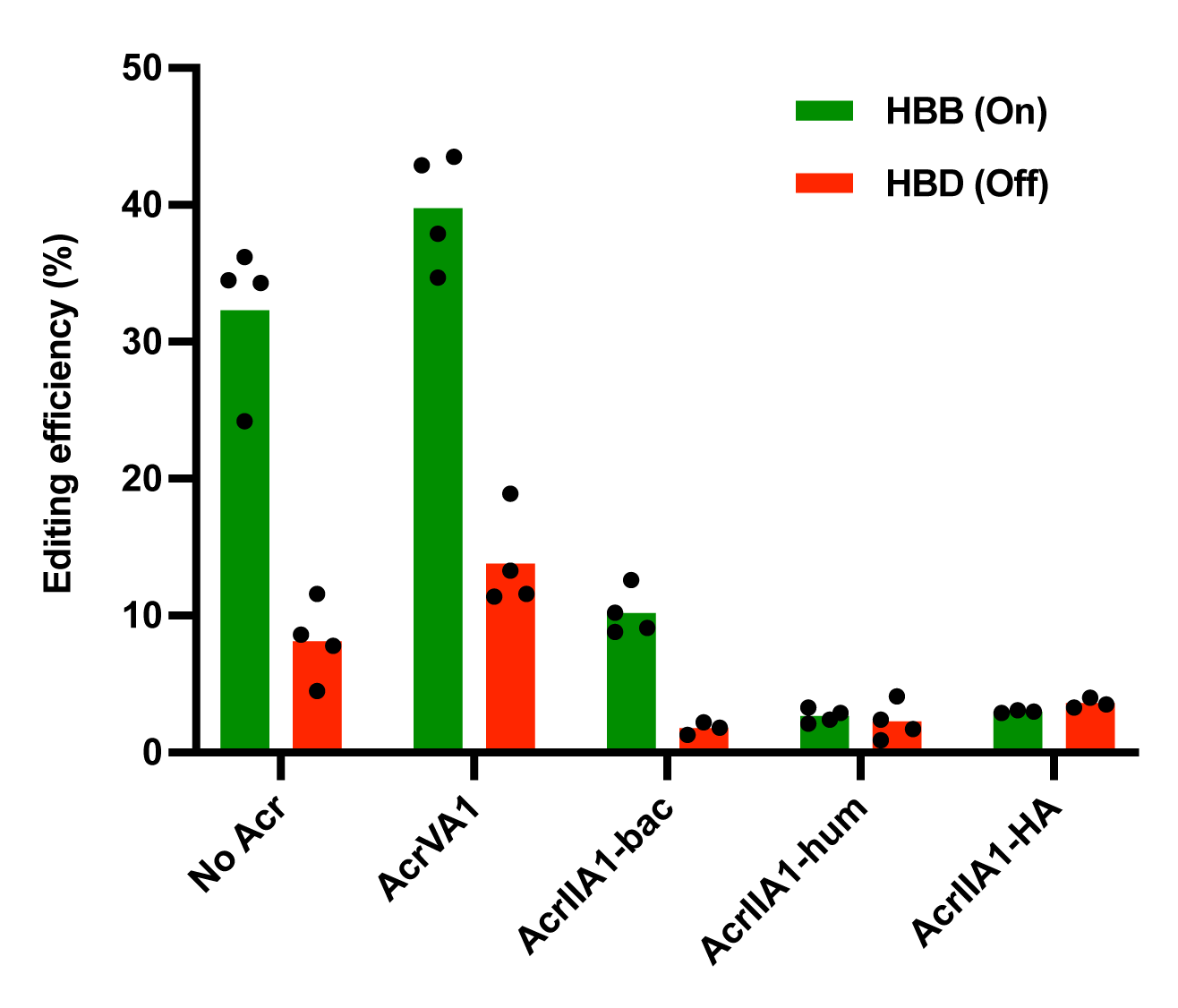

Supplement: S2 Fig — Editing by SpyCas9 of the HBB gene and the closely related off-target site HBD. AcrIIA1-bac uses the native bacterial codons. AcrIIA1-hum is codon optimized for human expression. AcrIIA1-HA is the AcrIIA1-hum with an HA tag. HEK293T cells were transiently transfected at a plasmid ratio of 1:2 SpyCas9:AcrIIA1 plasmid. No difference is observed between AcrIIA1-hum and AcrIIA1-HA (P = 0.35). Bars represent the mean of biological replicates (dots). Underlying data can be found in S1 Data. (TIF) [file pbio.3002431.s002.tif]

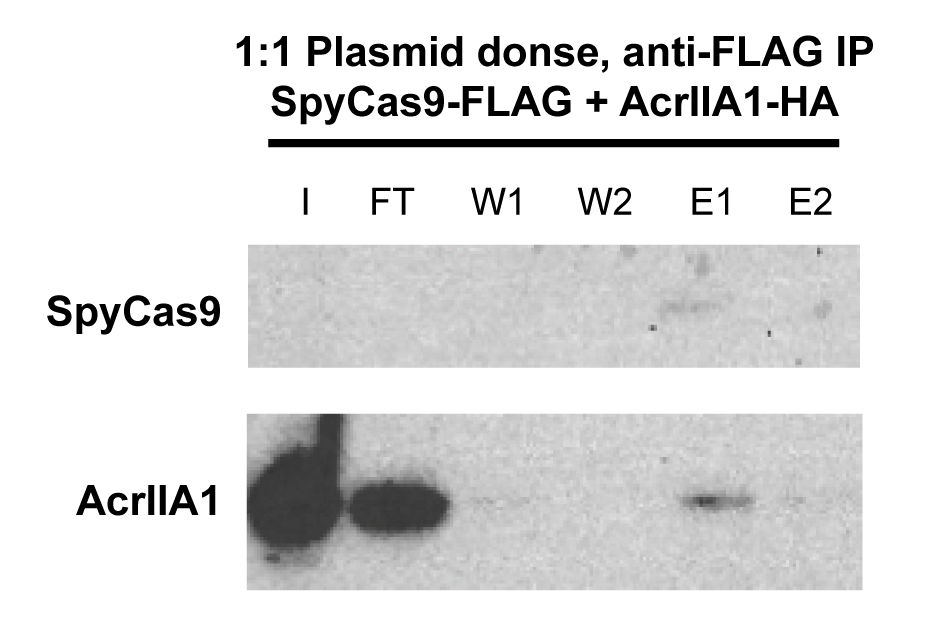

Supplement: S3 Fig — Western blot of anti-FLAG immunoprecipitations pulling down FLAG-tagged SpyCas9 and probing for SpyCas9 and AcrIIA1. Co-expression of SpyCas9-FLAG and AcrIIA1-HA (1:1 plasmid ratio). AcrIIA1 binds and elutes (E1) along with the residual SpyCas9. I = input, FT = flow through, W1 = wash 1, W2 = wash 2, E1 = elution 1, E2 = elution 2. (TIF) [file pbio.3002431.s003.tif]

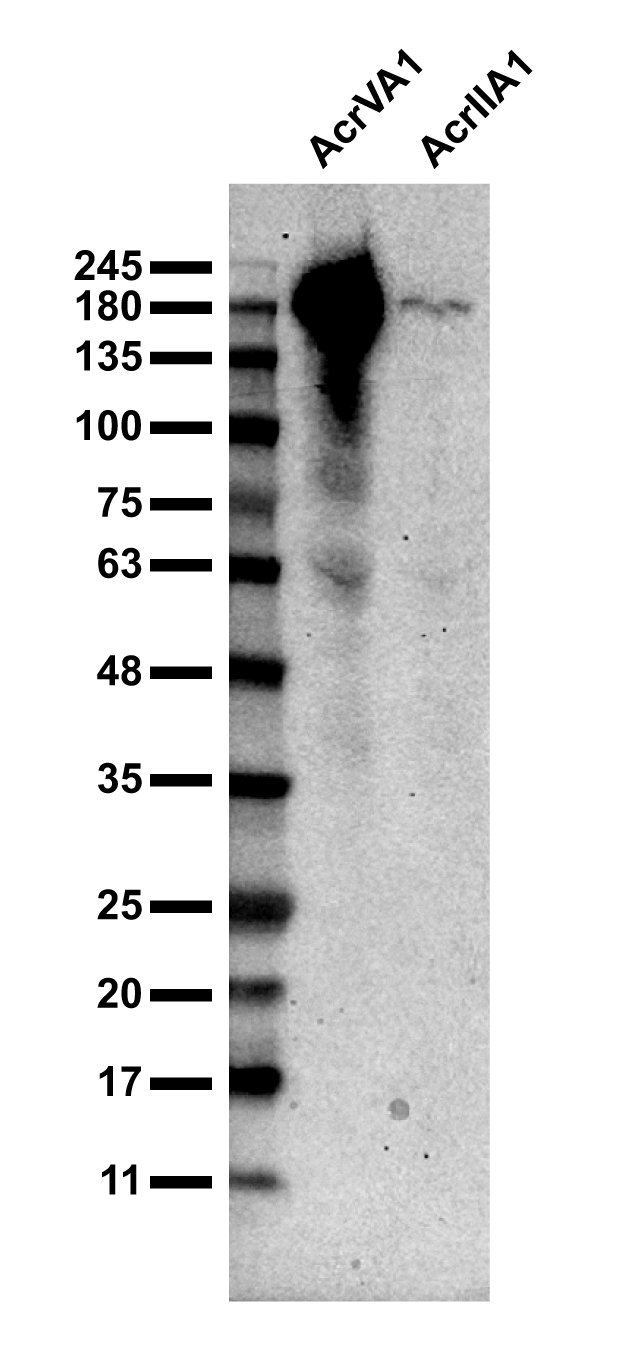

Supplement: S4 Fig — Western blot showing AcrIIA1-dependent decrease in SpyCas9 protein level in HEK293T cell lysates compared to AcrVA1. No degradation products are seen in the AcrIIA1 condition that are not present in the AcrVA1 lysate. SpyCas9 is detected using a monoclonal anti-SpyCas9 antibody. (TIF) [file pbio.3002431.s004.tif]
