# Supplementary material for: Cas9 degradation in human cells using phage anti-CRISPR proteins
Source: PLoS Biol. 2023 Dec 8;21(12):e3002431. doi: 10.1371/journal.pbio.3002431 (PMC10732428; doi:10.1371/journal.pbio.3002431)

**Supporting Information Raw Images:** Uncropped and minimally adjusted images for all relevant figures in this article. Areas of interest used in figures are outlined in a red box. Experimental approach, antibody, detection strategy, and equipment are indicated for each image. Sizes are shown corresponding to molecular weight markers for proteins. Lanes not used in the figure are marked with “X”.

Fig 2A (SpyCas9)

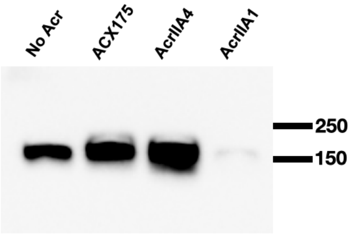


Fig 2A (SpyCas9)

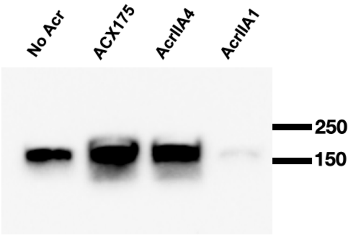


Fig 2A (SpyCas9)

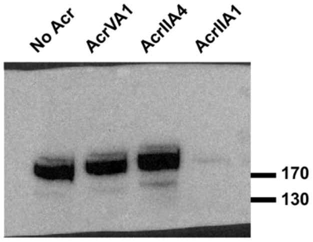


Fig 2A (HA)

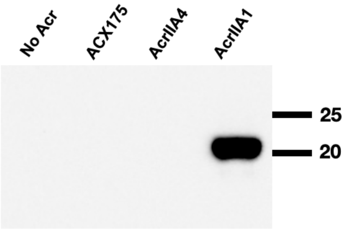


Fig 2A (HA)

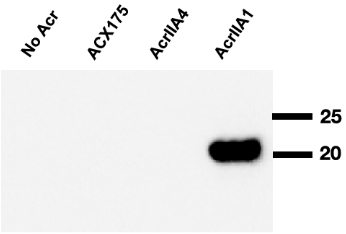


Fig 2A (HA)

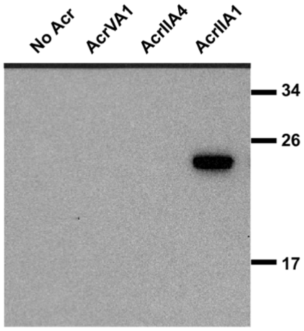


Fig 2A (HSP90)

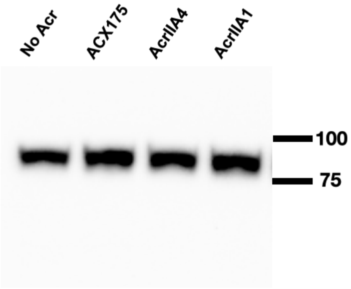


Fig 2A (HSP90)

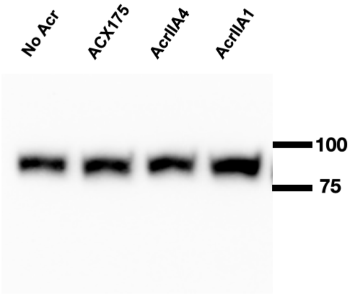


Fig 2A (HSP90)

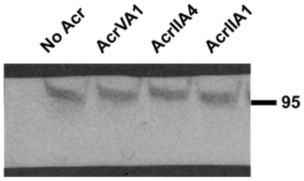

Fig 2B (SpyCas9)

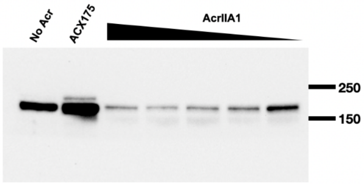


Fig 2B (SpyCas9)

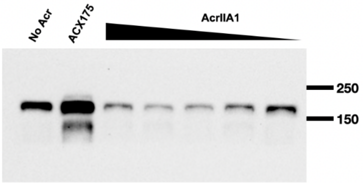


Fig 2B (SpyCas9)

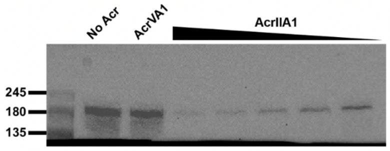


Fig 2B (HA)

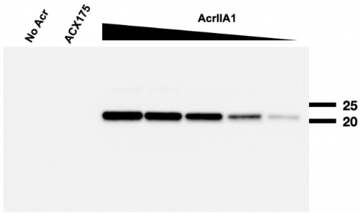


Fig 2B (HA)

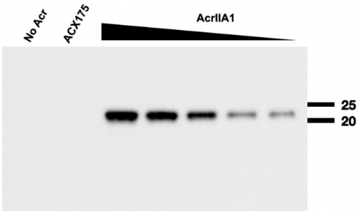


Fig 2B (HA)

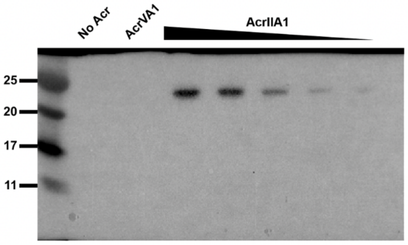


Fig 2B (HSP90)

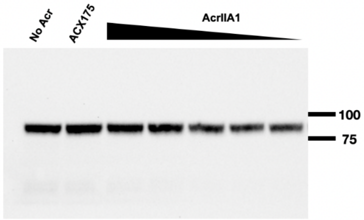


Fig 2B (HSP90)

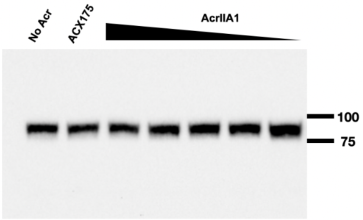


Fig 2B (HSP90)

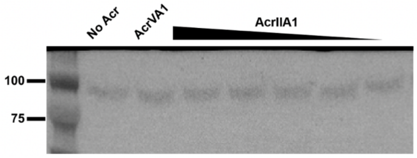

**Fig 2C (SpyCas9)**

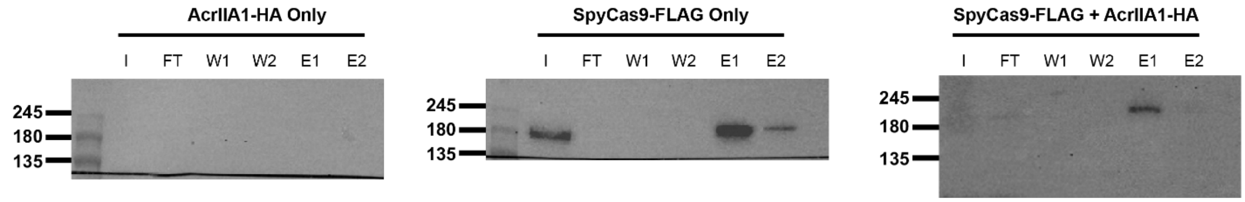


**Fig 2C (HSP90)**

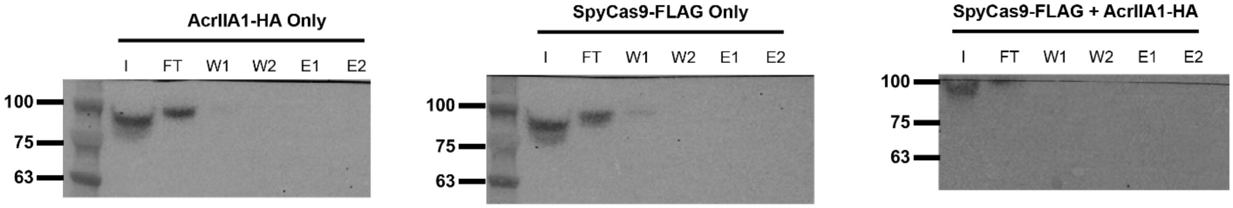


**Fig 2C (HA)**

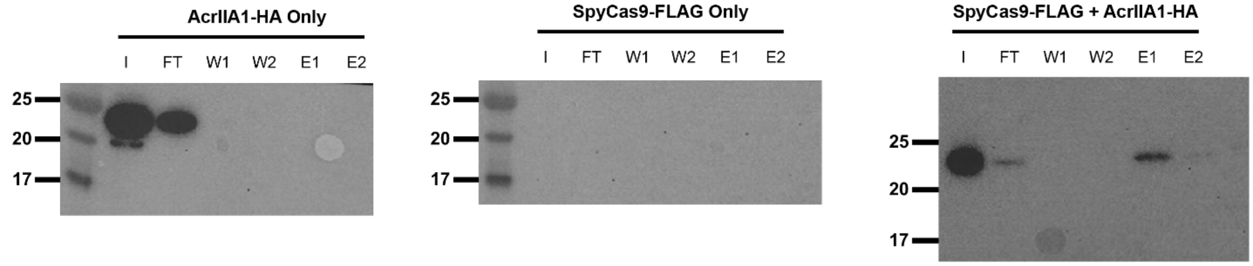

**Fig 2E (SpyCas9)**

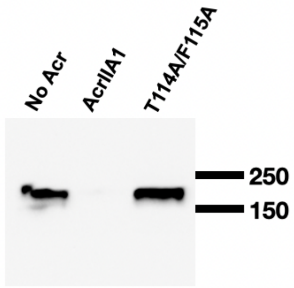


**Fig 2E (SpyCas9)**

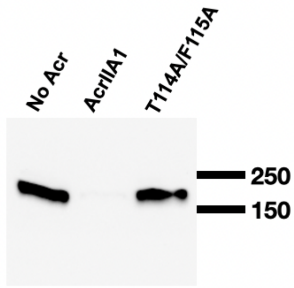


**Fig 2E (SpyCas9)**

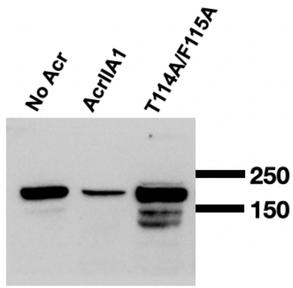


**Fig 2E (HA)**

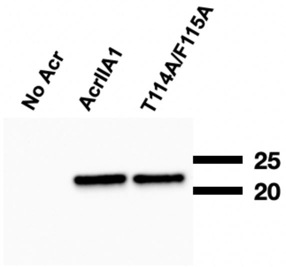


**Fig 2E (HA)**

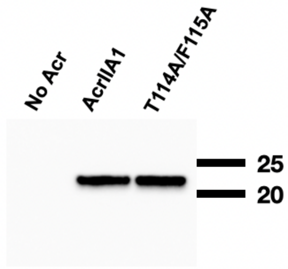


**Fig 2E (HA)**

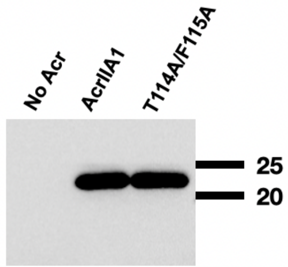


**Fig 2E (HSP90)**

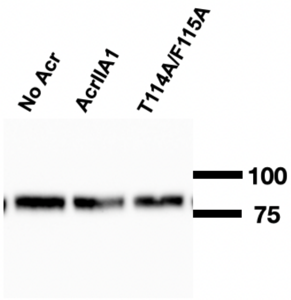


**Fig 2E (HSP90)**

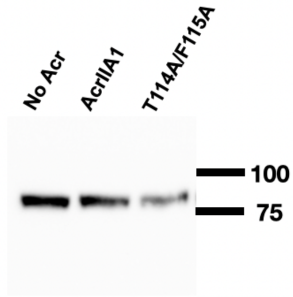


**Fig 2E (β-Actin)**

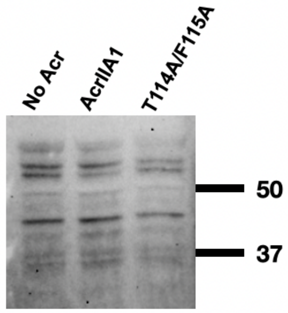

Fig 3B (SauCas9)

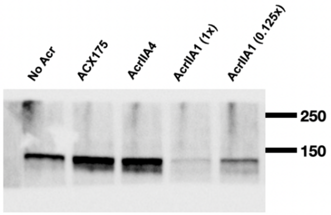


Fig 3B (SauCas9)

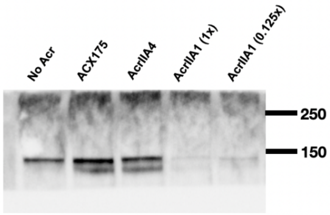


Fig 3B (SauCas9)

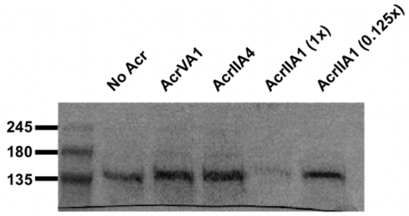


Fig 3B (HA)

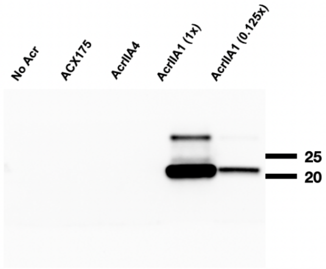


Fig 3B (HA)

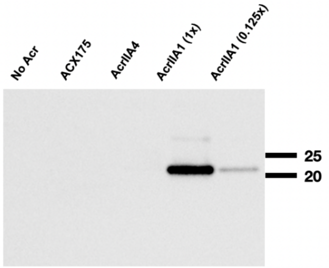


Fig 3B (HA)

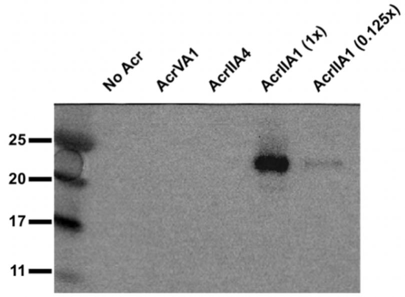


Fig 3B (HSP90)

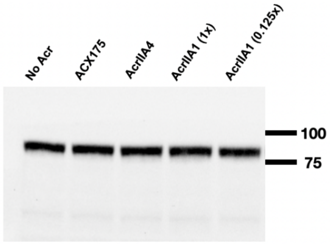


Fig 3B (HSP90)

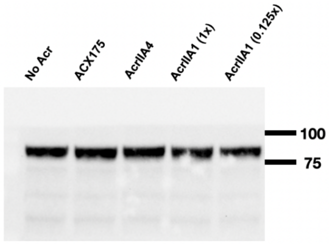


Fig 3B (HSP90)

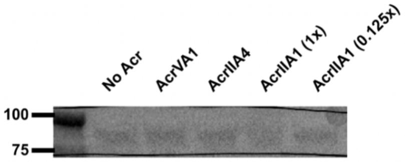

Fig 3C (AsCas12a)

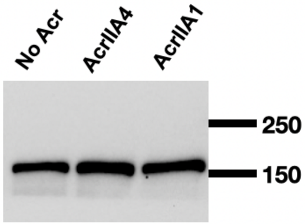


Fig 3C (AsCas12a)

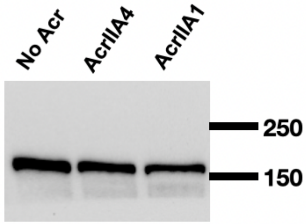


Fig 3B (AsCas12a)

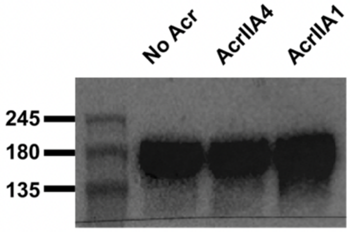


Fig 3C (HA)

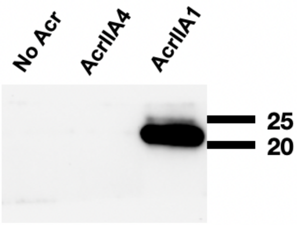


Fig 3C (HA)

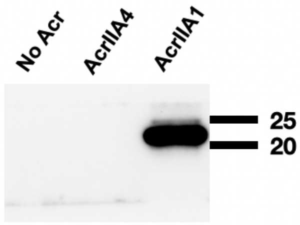


Fig 3C (HA)

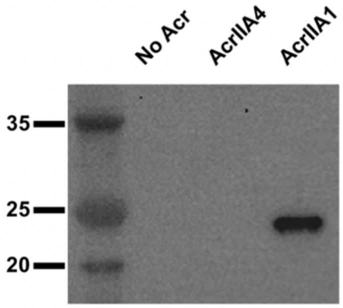


Fig 3C (HSP90)

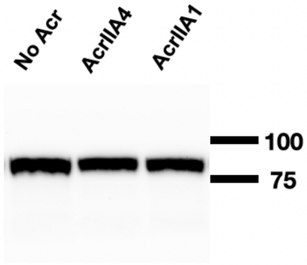


Fig 3C (HSP90)

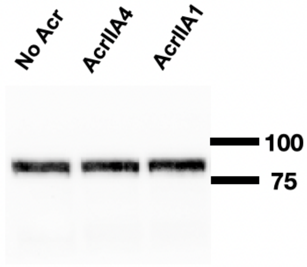


Fig 3C (HSP90)

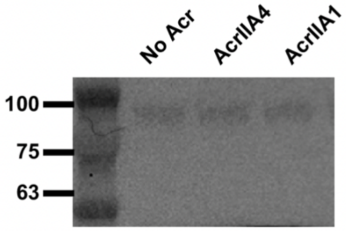

Fig S1B (SpyCas9)

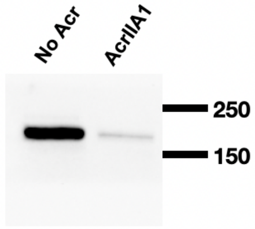


Fig S1B (SpyCas9)

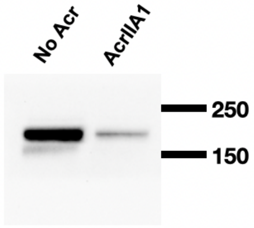


Fig S1B (SpyCas9)

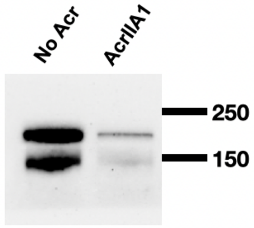


Fig S1B (HA)

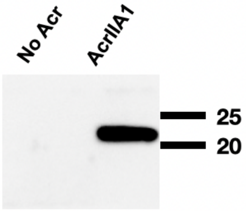


Fig S1B (HA)

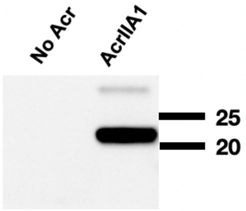


Fig S1B (HA)

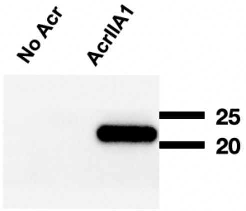


Fig S1B (HSP90)

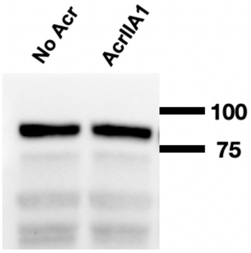


Fig S1B (HSP90)

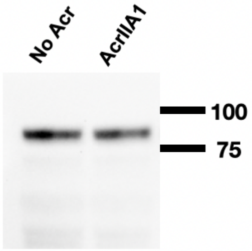


Fig S1B (HSP90)

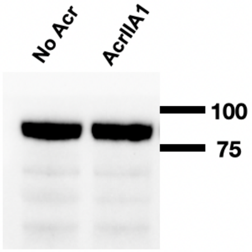

S3 Fig (SpyCas9)

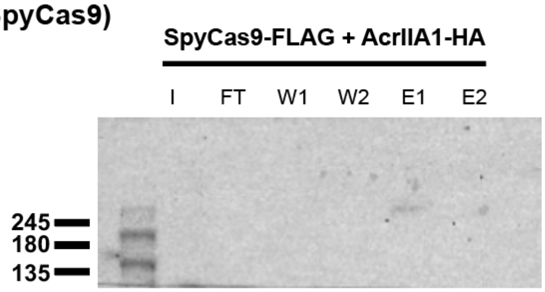


S3 Fig (HSP90)

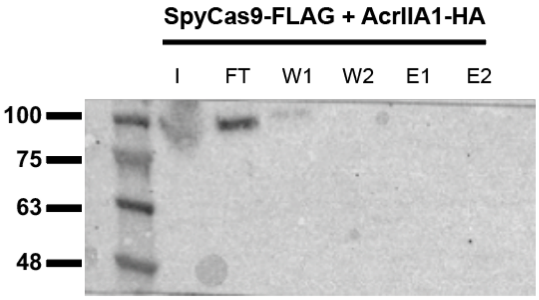


S3 Fig (HA)

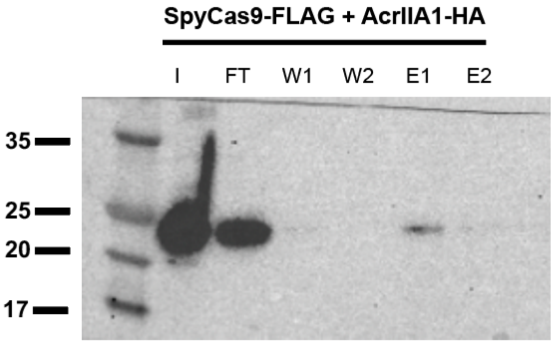

**S4 Fig. (SpyCas9)**

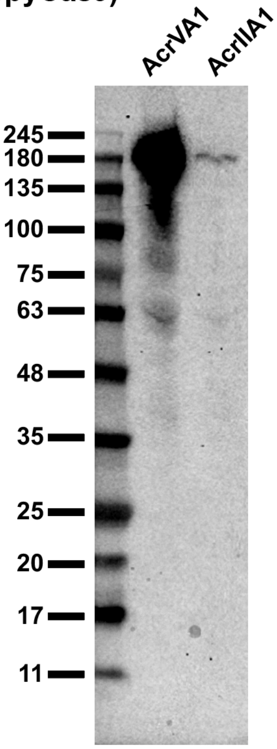

Supplement: S1 Raw Images — Areas of interest used in figures are outlined in a red box. Experimental approach, antibody, detection strategy, and equipment are indicated for each image. Sizes are shown corresponding to molecular weight markers for proteins. Lanes not used in the figure are marked with “X.” (PDF) [file pbio.3002431.s005.pdf]
